# Production of 3-Hydroxypropionic Acid via the Propionyl-CoA Pathway Using Recombinant *Escherichia coli* Strains

**DOI:** 10.1371/journal.pone.0156286

**Published:** 2016-05-26

**Authors:** Hui Luo, Dafeng Zhou, Xiaohui Liu, Zhihua Nie, Diego Leandro Quiroga-Sánchez, Yanhong Chang

**Affiliations:** 1 Department of Biological Science and Engineering, University of Science and Technology Beijing, Beijing, 100083, China; 2 Department of Environmental Engineering, University of Science and Technology Beijing, Beijing, 100083, China; 3 Key Laboratory of Educational Ministry for High Efficient Mining and Safety in Metal Mine, University of Science and Technology Beijing, Beijing, 100083, China; University of Freiburg, GERMANY

## Abstract

Our study aimed to produce the commercially promising platform chemical 3-hydroxypropionic acid (3-HP) via the propionyl-CoA pathway in genetically engineered *Escherichia coli*. Recombinant *E*. *coli Ec*-P overexpressing propionyl-CoA dehydrogenase (PACD, encoded by the *pacd* gene from *Candida rugosa*) under the T7 promoter produced 1.33 mM of 3-HP in a shake flask culture supplemented with 0.5% propionate. When propionate CoA-transferase (PCT, encoded by the *pct* gene from *Megasphaera elsdenii*) and 3-hydroxypropionyl-CoA dehydratase (HPCD, encoded by the *hpcd* gene from *Chloroflexus aurantiacus*) were expressed along with PACD, the 3-HP titer of the resulting *E*. *coli Ec*-PPH strain was improved by 6-fold. The effect of the cultivation conditions on the 3-HP yield from propionate in the *Ec*-PPH strain was also investigated. When cultured at 30°C with 1% glucose in addition to propionate, 3-HP production by *Ec*-PPH increased 2-fold and 12-fold compared to the cultivation at 37°C (4.23 mM) or without glucose (0.68 mM). Deletion of the *ygfH* gene encoding propionyl-CoA: succinate CoA-transferase from *Ec*-PPH (resulting in the strain *Ec*-*△Y*-PPH) led to increase of 3-HP production in shake flask experiments (15.04 mM), whereas the strain *Ec*-*△Y*-PPH with deletion of the *prpC* gene (encoding methylcitrate synthase in the methylcitrate cycle) produced 17.76 mM of 3-HP. The strain *Ec*-*△Y-△P*-PPH with both *ygfH* and *prpC* genes deleted produced 24.14 mM of 3-HP, thus showing an 18-fold increase in the 3-HP titer in compare to the strain *Ec*-P.

## Introduction

3-Hydroxypropionic acid (3-HP; C_3_H_6_O_3_; MW 90.08) is one of the most valuable platform chemicals due to its great potential in the synthesis of novel polymer materials and other derivatives, such as 1,3-propanediol, malonic acid, acrylonitrile, and acrylamide, all of which are used in large quantities in industry [1–3]. Compared to the present costly multi-step chemical processes, the biosynthesis of 3-HP by microorganisms is considerded to be an effective alternative for its mild operation conditions and reduced environmental loads [4, 5].

It was reported that 3-HP could be accumulated as an end product from glycerol, glucose or acrylic acid in a variety of microorganisms [6–8]. Several distinct enzymatic routes have been proposed for microbial 3-HP production [9–14], among them, the modified β-oxidation pathway (also known as the propionyl-CoA pathway: propionic acid → propionyl-CoA → acryloyl-CoA → 3-hydroxypropionyl-CoA → 3-HP) that likely exists in some microorganisms that naturally produce 3-HP [15]. In *E*. *coli* grown on glucose propionyl-CoA can be derived from succinyl-CoA [16], and a *Candida rugosa* mutant is able to produce 40.2 g/l of 3-HP, presumably via the propionyl-CoA pathway. This is the highest titer obtained from non-engineered microorganisms in the literature. This route is therefore proposed to have great potential for the bioengineering of *E*. *coli* to produce 3-HP from propionyl-CoA pathway [15]. However, the biological production of 3-HP via the propionyl-CoA pathway in *E*. *coli* has not been studied.

**Fig 1:**
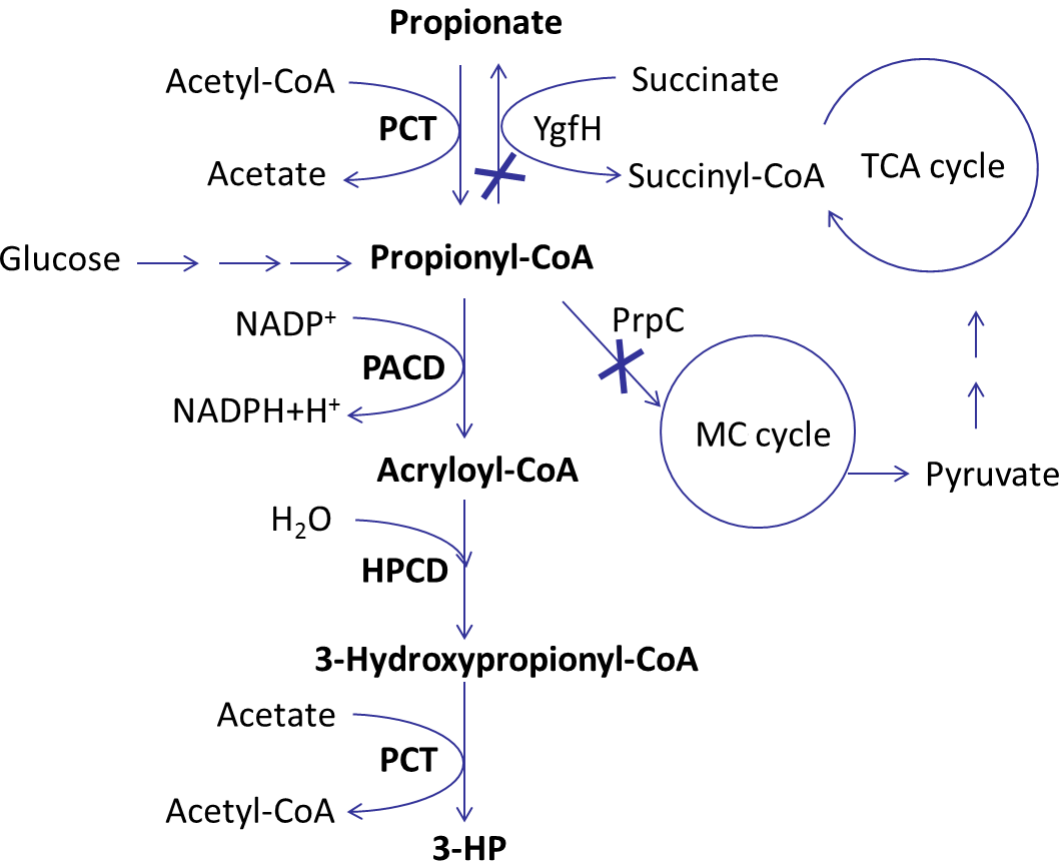
Metabolic pathway involved in the conversion of propionate to 3-hydroxypropionate in recombinant *E*. *coli* strains. The enzymes in bold are overexpressed, while disrupted pathway steps are indicated by the bold “×” symbols. MC cycle: methylcitrate cycle; TCA cycle: tricarboxylic acid cycle; PCT: propionate CoA-transferase; PACD: propionyl-CoA dehydrogenase; HPCD: 3-hydroxypropionyl-CoA dehydratase; YgfH: propionyl-CoA: succinate-CoA transferase; PrpC: methylcitrate synthase.

Only a few key enzymes need to be introduced to establish 3-HP production via the propionyl-CoA pathway in *E*. *coli* (Fig 1). These enzymes are propionate CoA-transferase (PCT, EC:2.8.3.1) [17–19], catalyzing the CoA transfer from acetyl-CoA to propionate, propionyl-CoA dehydrogenase (PACD, EC:1.3.8.7) [15, 20, 21], which subsequently converts propionyl-CoA to acryloyl-CoA, and 3-hydroxypropionyl-CoA dehydratase (HPCD, EC:4.2.1.17) [22, 23], converting acryloyl-CoA to 3-hydroxypropionyl-CoA. Finally, 3-hydroxypropionyl-CoA (3-HP-CoA) can be converted to 3-HP by the action of either PCT or 3-hydroxypropionyl-CoA hydrolase [17, 18]. In this schematic biosynthesis of 3-HP, competitive pathways such as the methylcitrate cycle (MC cycle) and tricarboxylic acid cycle (TCA cycle) are also indicated (Fig 1).

Propionate and its derivative propionyl-CoA can be metabolized by *E*. *coli* (propionyl-CoA metabolism pathway in KEGG Database: pathway ID ko00640) [24]. To increase the yield of 3-HP on propionate, these competing propionate metabolic pathways should be down-regulated or even disrupted. Enzymes such as propionyl-CoA: succinate CoA-transferase (YgfH) [25, 26] and methylcitrate synthase (PrpC) [24, 27, 28] are responsible for the propionate metabolism in *E*. *coli*, and deleting them could result in an increased yield of 3-HP [26, 28, 29].

In this work, the genes of PCT, HPCD and PACD were obtained from different bacterial species, and a recombinant *E*. *coli* containing the propionyl-CoA pathway for 3-HP production was constructed. The effects of the culture conditions (e.g., temperature and carbon source) on 3-HP accumulation were also tested. To improve the 3-HP yield on propionate by preventing the degradation of propionyl-CoA in the cells, the *ygfH* and *prpC* genes in the host strain were deleted, and the 3-HP productivity of the engineered strains was evaluated.

## Materials and Methods

### Materials

The genomic DNA isolation kit and the TA cloning vector pBS-T were purchased from Tiangen Co., Ltd. (Beijing, China). The RNApure yeast kit and cDNA synthesis kit were purchased from Aidlab Biotechnologies Co., Ltd. (Beijing, China). The DNA gel purification kit was purchased from Promega (USA). The restriction and DNA-modifying enzymes were obtained from Takara Co., Ltd. (Dalian, China). The primers were synthesized by Sunbiotech Co., Ltd. (Beijing, China). 3-HP was purchased from Tokyo Chemical Industry Co., Ltd. Propionic acid and all other chemicals were purchased from Biodee Co., Ltd. (Beijing, China) as analytical grade.

### Cloning of *pct*, *pacd* and *hpcd* genes

**Fig 2:**
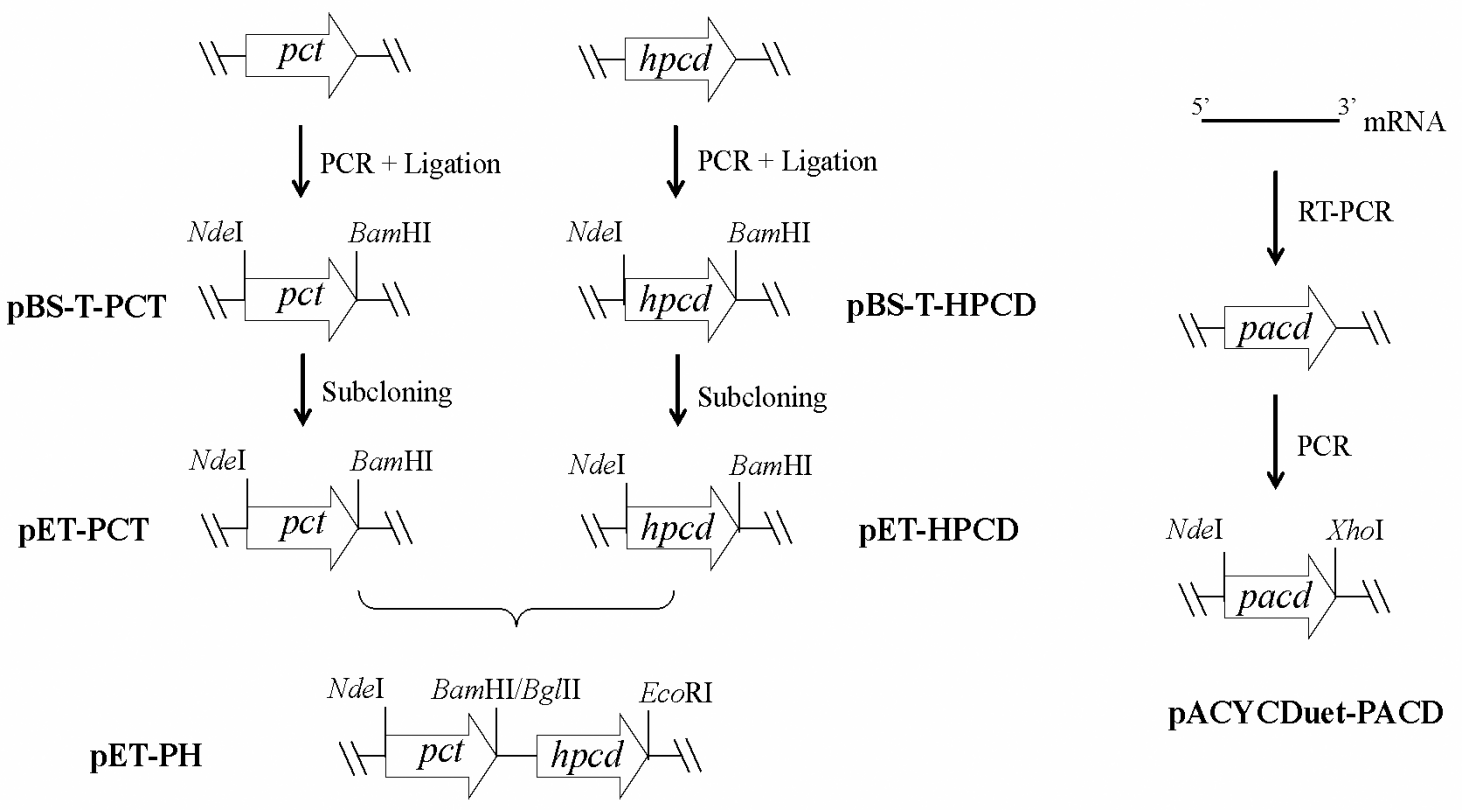
Construction of plasmids pET-PH and pACYCDuet-PACD.

The bacterial strains and the plasmids used in this study are summarized in Table 1. Lysogeny broth (LB) medium was used for the routine genetic engineering, protein expression and culture maintenance. A final concentration of 50 mg/l kanamycin and 25 mg/l chloramphenicol were added to the medium before inoculation. Gene manipulations were carried out using standard methods [30].To express *pacd*, the pACYCDuet-1 vector with the T7 promoter was adopted. The *pct* and *hpcd* genes were cloned into the pET-28a vector. Details on the construction of the plasmids are shown in Fig 2.

**Table 1:** Bacterial strains and plasmids used in the study.

Strains and plasmids	Description	Source
Strains		
*Candida rugosa*	Source for *pacd*	CICC, China
*Megasphaera elsdenii*	Source for *pct*	CICC, China
*Choroflexus aurantiacus*	Source for HPCD gene *hpcd*	CAS, China
*E*. *coli* TOP 10	Cloning host	Novagen
*E*. *coli* BL21(DE3)	Expression host	Novagen
*E*. *coli* JM109(DE3)	Expression host	Tiangen
*Ec-△Y*	*E*. *coli* JM109(DE3) *△ygfH*	This study
*Ec-△P*	*E*. *coli* JM109(DE3) *△prpC*	This study
*Ec-△Y-△P*	*E*. *coli* JM109(DE3) *△ygfH*,*△prpC*	This study
*Ec*-P	Recombinant *E*. *coli* BL21(DE3) harboring plasmid pACYCDuet-PACD	This study
*Ec*-PPH	Recombinant *E*. *coli* BL21(DE3) harboring plasmid pACYCDuet-PACD and pET-PH	This study
*Ec-△Y*-PPH	Recombinant *E*. *coli* JM109(DE3) *△ygfH* harboring plasmid pACYCDuet-PACD and pET-PH	This study
*Ec-△P*-PPH	Recombinant *E*. *coli* JM109(DE3) *△prpC* harboring plasmid pACYCDuet-PACD and pET-PH	This study
*Ec-△Y-△P*-PPH	Recombinant *E*. *coli* JM109(DE3) *△ygfH*,*△prpC* harboring plasmid pACYCDuet-PACD and pET-PH	This study
Plasmids		
pBS-T	*lacZa*, TA cloning vector, T7/T3 promoter, Amp^r^	Tiangen
pET-28a	*lacI*, expression vector, T7 promoter, PBR322-ori, kanamycin (Kan)^r^	Novagen
pACYCDuet-1	*lacI*, expression vector, T7 promoter, P15A-ori; 2 sets of MCS, MCS I-His_6_-N, MCS II-S-tag-N, chloramphenicol (Cm)^r^	Novagen
pKD46	repA101(Ts), ara-gamma-Beta-exo, onR101, ampicillin(Amp)^r^	Reference[31]
pKD13	FTP, Kan^r^	Reference[31]
pCP20	Rep(Ts), cI857λ(Ts), Amp^r^, Cm^r^, FLP	Reference[31]
pBS-T-PCT	*pct* in pBS-T vector, Amp^r^	This study
pBS-T-HPCD	*hpcd* in pBS-T vector, Amp^r^	This study
pET- PCT	*pct* in pET28a vector, Kan^r^	This study
pET-HPCD	*hpcd* in pET28a vector, Kan^r^	This study
pET-PH	*pct* and *hpcd* in pET28a vector, Kan ^r^	This study
pACYCDuet-PACD	*pacd* in pACYCDuet-1 vector, Cm^r^	This study

To construct a recombinant expression vector pACYCDuet-PACD, the full-length cDNA encoding the PACD of *Candida rugosa* was cloned by RT-PCR using the Aidlab Biotechnologies kit according to the manufacturer’s instructions, and the coding region of PACD (GenBank number GU338397) was amplified by PCR with the primers listed in S1 Table. The *Nde* I, *Xho* I-digested PCR product was finally incorporated into the MCS-II of the pACYCDuet-1 vector.

The *pct* gene (GenBank: HE576794.1) was obtained from the isolated genomic DNA of *Megasphaera elsdenii* by PCR. The 1554 bp PCR fragment was first ligated in the pBS-T vector by means of TA cloning, and its sequence was confirmed by Sunbiotech Co., Ltd (Beijing). The *pct* gene was then released by the flanking *Nde*I and *Bam*HI sites and sub-cloned into the pET-28a vector to generate pET-PCT.

To obtain the HPCD-overexpressing plasmid pET-HPCD, the *hpcd* gene (encoding HPCD) was amplified from the genomic DNA of *Chloroflexus aurantiacus* and successively constructed in the pBS-T and pET-28a vectors using the same method as for *pct*. The T7-HPCD fragment was then digested via *Bgl*II and *EcoR*I sites from pET-HPCD, and the pET-PCT fragment missing the sequence between the *Bam*HI (the isocaudomer of *Bgl*II) and *EcoR*I sites was incorporated with it, thereby obtaining pET-PH.

The plasmid pACYCDuet-PACD was transformed into *E*. *coli* BL21(DE3) individually and combined with pET-PH, and the recombinant strains *Ec*-P and *Ec*-PPH were obtained for protein expression and 3-HP production, respectively.

### Gene deletion of YgfH and PrpC

The *ygfH* and *prpC* gene deletions of *Ec*-PPH were performed by directly disrupting the target gene via kanamycin marker insertion using Red recombination, followed by the kanamycin resistance cassette excision mediated through the flippase recombination enzyme harbored by pCP20 [31]. The primers used for inserting the kanamycin cassette into the *ygfH* and *prpC* genes are listed in S1 Table. The *ygfH*, *prpC*, and both *ygfH* and *prpC* gene deletion mutants are referred to as *Ec*-△Y-PPH, *Ec*-△P-PPH, and *Ec*-△Y-△P -PPH, respectively.

### Protein expression and gel electrophoresis

To verify that the recombinant strains had been properly constructed, the parent strain *E*. *coli* BL21(DE3) and two recombinant strains (*Ec*-P and *Ec*-PPH) were cultured in LB medium and induced at 25°C with 50 μM IPTG at OD_600_ of 0.8. The cells were harvested after 6 h induction and centrifuged at 10,000 g at 4°C for 15 min. The cell pellets were washed twice and then resuspended in 100 mM potassium phosphate buffer (pH 7.0). The cells were lysed by sonication and then centrifuged at 10,000 g for 20 min. The supernatants were subjected to SDS-PAGE to examine protein expression [30]. Coomassie Brilliant Blue R250 was used for protein staining.

### Cultivation of recombinant *E*. *coli* strains

Unless indicated otherwise, shake flask cultivation was carried out with a 20 mL working volume in 100-ml Erlenmeyer flasks at 30°C in an incubator shaker at 200 rpm. The media used in this study were MI medium, containing glucose 1%, peptone 1%, yeast extract 0.5%, NaCl 1% and MgSO_4_·7H_2_O 0.025%, pH 7.0, and MII medium, which is MI medium without the glucose. Kanamycin (Kan) at 50 mg/l was added to the strain *Ec*-P. For *E*c-PPH, kanamycin and chloramphenicol (Cm) were added at 50 mg/l and 25 mg/l, respectively. The recombinant strains were cultured in the above mediums and induced at OD_600_ of 0.8 with 50 μM IPTG. After 6 h induction, 0.5% propionate was added. Samples were withdrawn periodically to determine the concentrations of 3-HP and residual propionate.

### Analytical methods

The propionate and 3-HP concentrations were assayed by high performance liquid chromatography (HPLC). The supernatants obtained by the centrifugation of the culture samples at 10,000 g for 10 min were filtered through a 0.22 μm filter and isocratically eluted through a Venusil MP C18 column (4.6 mm×250 mm, 5 μm particle size, Agela Technologies, Inc.) with methanol-10 mM H_3_PO_4_ (5:95, v/v) as the mobile phase_._ The flow rate and the detection wavelength were set to 0.8 ml/min and UV 208 nm, respectively. Pure 3-HP and propionate were assayed by HPLC independently to determine their retention time to be able to identify them later in the chromatogram of each sample. The samples of three independent cultures were assayed, and the standard deviation of the measurements was less than 5% for propionate and 3-HP.

## Results

### Expression of PCT, HPCD and PACD in recombinant *E*. *coli*

To construct a pathway as shown in Fig 1, three enzymes corresponding to the formation of the intermediates propionyl-CoA, acryloyl-CoA and 3-hydroxypropionyl-CoA are required.

*E*. *coli* possesses genes encoding PrpE (propionyl-CoA synthetase) [32, 33] and enoyl-CoA hydratase [34, 35], which are functionally similar to PCT and HPCD, respectively. It does, however, lack a gene encoding PACD. Therefore, introducing PACD alone may already be enough to establish the pathway in *E*. *coli*.

It was reported that the overexpression of propionyl-CoA synthetase (PrpE) in *E*. *coli* could increase the formation of propionyl-CoA [28]. However, the activation of propionate to its CoA ester by PrpE requires two ATPs [35]. Thus, employing a CoA transferase could be preferable for the production of propionyl-CoA, as no additional ATP would be required (Fig 1).

Enoyl-CoA hydratase catalyzes the hydration of α,β-unsaturated CoA thioesters during ß-oxidation of fatty acids [36]. There might be low substrate specificity for the enoyl-CoA hydratase of *E*. *coli* for acryloyl-CoA, which is not a native metabolite in the host cell. Hence, the gene *hpcd* from *Chloroflexus aurantiacus* [22], encoding an enoyl-CoA hydratase specific for acryloyl-CoA, was cloned and expressed in the recombinant *E*. *coli*.

**Fig 3:**
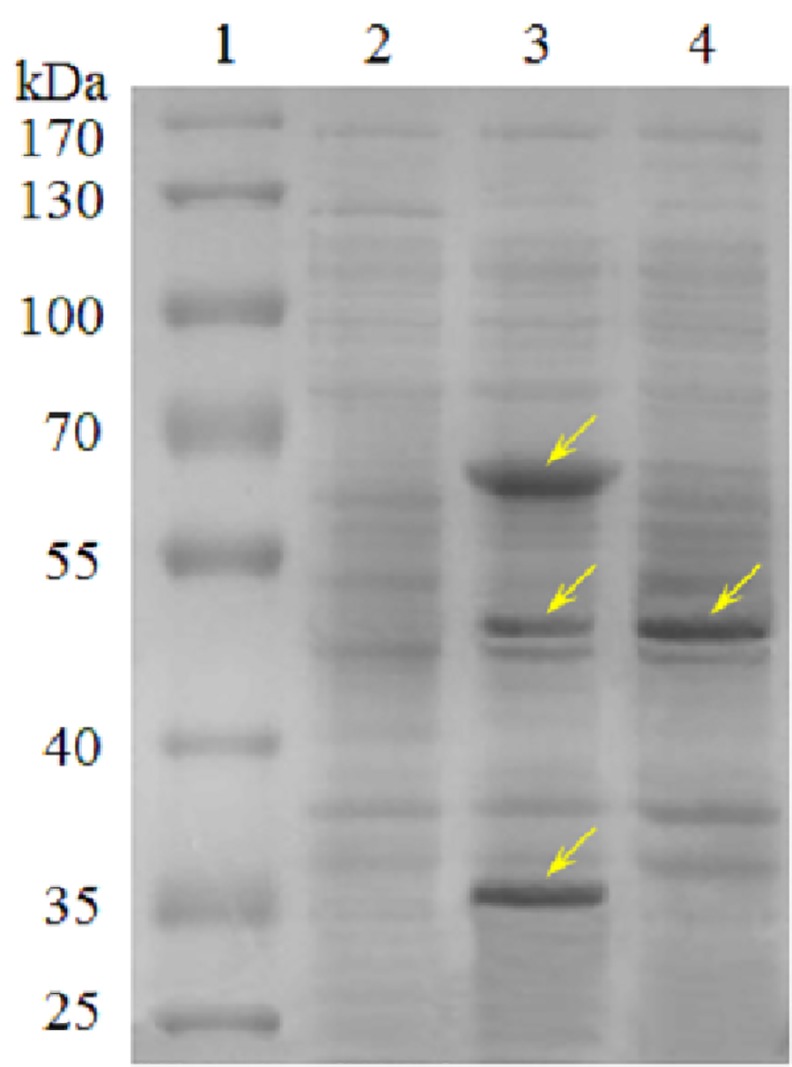
SDS-PAGE analysis of cell extract of the host *E*. *coli*, *Ec*-PPH and *Ec*-P. (Lane 1) protein standard, (Lane 2) host *E*. *coli*, (Lane 3) *Ec*-PPH expressing PACD, PCT and HPCD, (Lane 4) *Ec*-P with PACD expression alone. The arrow marks indicate the corresponding target proteins.

Therefore, to produce 3-HP via the propionyl-CoA pathway, the recombinant *E*. *coli* strains *Ec*-PPH (expressing PACD, PCT and HPCD) and *Ec*-P (expressing PACD alone) were constructed, and the protein expressions of these strains were assayed. Fig 3 shows the expression profile of the desired recombinant proteins. The heterologous genes of PCT, PACD and HPCD could be expressed in a soluble form in the recombinant *E*. *coli*. PACD (with a molecular weight of 49 kDa) was found to be the most abundant protein in the cell lysate of *Ec*-P. When PACD was co-expressed with PCT and HPCD in *Ec*-PPH, its expression level decreased to some extent (Fig 3).

### Cultivation of the recombinant *E*. *coli* strains for 3-HP production

**Fig 4:**
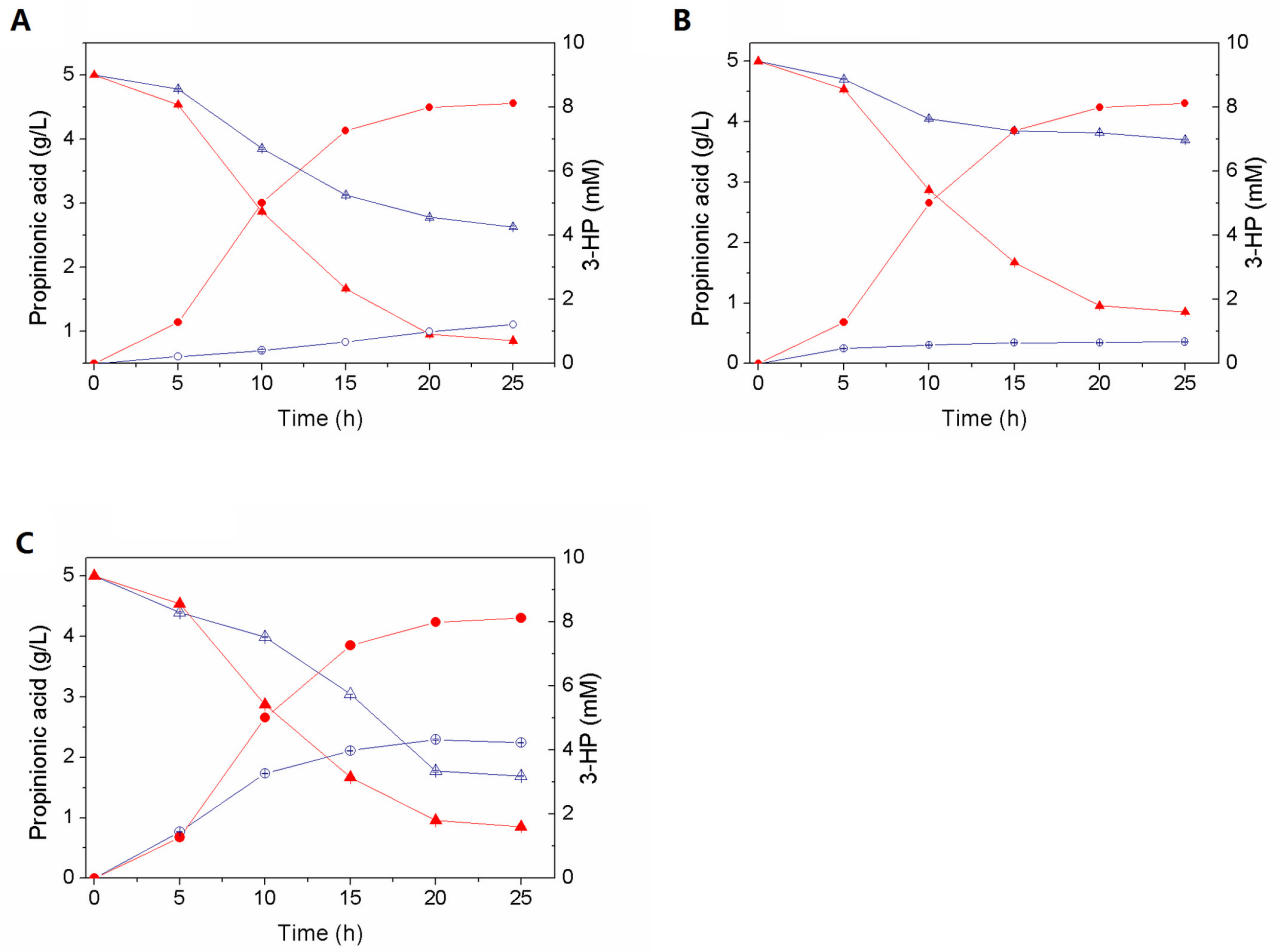
Time-course profile of propionic acid consumption and production of 3-HP for two strains, Ec-PPH and Ec-P. (A) *Ec*-PPH (expressing PACD PCT and HPCD, closed symbols) and *Ec*-P (expressing PACD, open symbols) were cultured in MI medium at 30°C. (B) *Ec*-PPH was cultivated in medium with glucose (1%, closed symbols) and medium without glucose (open symbols) at 30°C. (C) *Ec*-PPH cultivation in MI medium at 30°C (closed symbols) and 37°C (open symbols). Symbols: 3-HP (circle), propionic acid (triangle). The experiment was done in triplicate. The error bars show standard errors and are not shown if the error did not exceed the size of the symbol.

To evaluate the potential of the recombinant strains for the production of 3-HP through the propionyl-CoA pathway, the cultivation of *EC*-P and *Ec*-PPH was conducted in MI medium with 0.5% propionic acid at 30°C (Fig 4A). When PACD is expressed alone (*Ec*-P), the final 3-HP titer at 25 h was 1.33 mM. In comparison, when heterologous PCT and HPCD were co-expressed with PACD (*Ec*-PPH), the yield of 3-HP was increased 6-fold to 8.11 mM.

As mentioned, propionyl-CoA utilizing metabolic pathways exist in *E*. *coli* [24, 26, 37], which lead to a reduced amount of propionyl-CoA being converted to acryloyl-CoA. Furthermore, in contrast to the low *K*_m_ value of some *E*.*coli* enzymes to propionyl-CoA (for example, the *K*_m_ value of PrpC to propionyl-CoA is 17 μM) [24], the *K*_m_ value of PACD to propionyl-CoA was reported to be as high as 40.86 μM [21]. Therefore, improvement of the PACD activity is necessary for efficient functioning of the modified β-oxidation pathway. On the other hand, inactivation of the *prpC* gene of the host should be helpful to increase the pool of intracellular propionyl-CoA, and the result of this experiment will be stated later in this work.

The production of 3-HP by *EC*-P overexpressing PACD alone indicates the presence of the propionyl-CoA synthetase and 3-hydroxypropionyl-CoA dehydratase activities. In the past decades, distinctive types of CoA transferase have been reported in many bacterial strains. Some of these enzymes have been purified from *Escherichia coli* [38], *Peptostreptococcus elsdenii* [18], *Ralstonia eutropha* [39], *Clostridium propionicum* [40] and *Clostridium sp*. strain SB4 [41] and some of them show catalytic activity for the conversion of propionate to propionyl-CoA. In addition, the 3-hydroxypropionyl-CoA dehydratase activities of various hydroxyacyl-CoA dehydratases have been demonstrated to be present in *Escherichia coli* [42] and several other species such as *Metallosphaera sedula* [43] *Sulfolobus tokodaii* [44] and *Nitrosopumilus maritimus* [45].

Compared to *Ec*-P, *Ec*-PPH showed a much higher 3-HP yield, which might be due to the high catalytic efficiencies of the co-expressed enzymes, PCT and HPCD. The overexpression of PCT in *Ec*-PPH was expected to increase the intracellular level of propionyl-CoA, which is an immediate substrate for PACD. The synthesis of propionyl-CoA by PCT does not need ATP, while the production of propionyl-CoA from propionate in *Ec*-P requires the function of its endogenous PrpE, which requires additional ATP [35]. The overexpression of HPCD in *Ec*-PPH could accelerate the consumption of the PACD product, namely acryloyl-CoA, leading to the increasd 3-HP yield.

The co-expression of heterologous PCT and HPCD helps to strengthen the desired pathway and reduce the generation of by-products. The results in Fig 4A suggest that to promote 3-HP production via the propionyl-CoA pathway, the PACD activity as well as the propionyl-CoA concentration and acryloyl-CoA conversion in the recombinant cells should be increased.

Glucose, a low-cost and highly efficient substrate, was considered in this work as an additional carbon source that may accelerate the 3-HP synthesis by *Ec*-PPH (Fig 4B).

In the absence of glucose, the production of 3-HP was only 0.67 mM, which is presumably owing to the insufficient ATP supply. As reported in Ref. 46, the synthesis of plasmid-encoded proteins is energetically demanding and leads to a redirection of metabolic fluxes in the cell, e.g. to an increased flow through the TCA cycle [46]. Without the glucose supplementation, propionic acid tends to be used for energy production, thus decreasing the flow through the propionyl-CoA pathway. However, propionic acid is not a preferable energy source as glucose is for *E*. *coli*, and ATP generated from propionate is far from being enough for the synthesis of growth-related and heterologous proteins, further weakening the 3-HP production.

The addition of glucose fulfilled the rising energy demands and resulted in a 12-fold increase in the 3-HP yield. As demonstrated here, the presence of glucose or another carbon source is required for the highly efficient production of 3-HP.

A previous study [21] reported that the PACD activity was the highest at 30°C. Nevertheless, it decreased to 30% of its original activity when incubated for 2 h at 37°C. At contrast, two other enzymes of the pathway (PCT, HPCD) had higher activity at 37°C, and this temperature is also optimal for *E*. *coli* [18]. Therefore, We compared the 3-HP production by *Ec*-PPH at 30 and 37°C.

As shown in Fig 4C, *Ec*-PPH produced only 4.23 mM 3-HP at 37°C (with the 3-HP yield from propionate of 8.2%), whereas 8.11mM 3-HP synthesized at 30°C (with the 14.5% yield). It suggests a crucial role of heterologous PACD in the process, thus revealing propionyl-CoA conversion to acryloyl-CoA as a rate-limiting step of the constructed pathway. In contrast, an increase of the temperature to 37°C (which is optimal for *E*. *coli* metabolism) results in increased by-products formation, weakening desirable 3-HP accumulation.

### Effect of *ygfH* and *prpC* deletion on 3-HP production

*E*. *coli* is known to be capable to metabolize propionyl-CoA [24, 26, 37]. For example, in the presence of succinate, propionyl-CoA can be converted by CoA-transferase into propionate and succinyl-CoA, and the succinyl-CoA is then utilized in the TCA cycle [26]. Propionyl-CoA could also be converted via the MC cycle to pyruvate, a key intermediate in cellular metabolism [24]. Thus, native *E*. *coli* metabolism of propionyl-CoA would result in a reduced amount of propionyl-CoA being converted to acryloyl-CoA.

**Fig 5:**
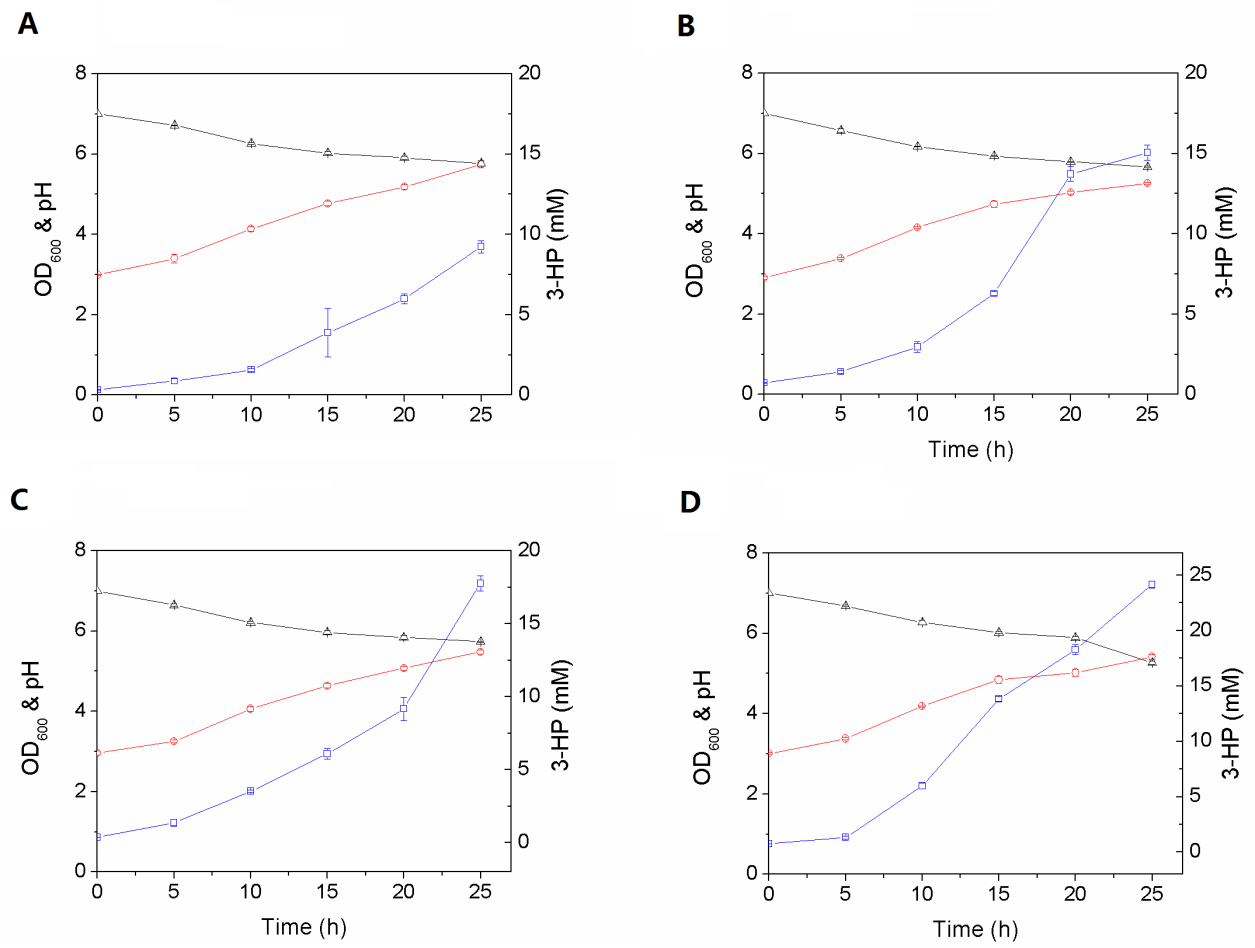
Production of 3-HP by recombinant *E*. *coli* deletion mutants expressing *pacd*, *pct* and *hpcd*. (A) WT *Ec*, (B) *Ec-ΔygfH*, (C) *Ec-ΔprpC*, and (D) *Ec-ΔygfH ΔprpC*. Symbols: 3-HP (square), cell mass biomass (circle), pH (triangle). The error bars denote standard errors of the mean from triplicate flasks and are not shown where the error did not exceed the size of the symbol.

As propionyl-CoA is a key precursor for 3-HP formation, the *E*. *coli* strain was further engineered to supply more propionyl-CoA into the 3-HP biosynthesis pathway by disrupting the competing metabolic pathways. 3-HP production from propionic acid was examined on a flask scale using four engineered *E*. *coli* host strains (wild type *E*. *coli*, *Ec-ΔygfH*, *Ec-ΔprpC*, and *Ec-ΔygfH ΔprpC*) containing *pacd*, *pct* and *hpcd* (Fig 5); they were tested three times independently.

As shown in Fig 5, the growth of all these strains was very similar, whereas the deletion of the *ygfH* and/or *prpC* genes significantly increased the 3-HP accumulation in the engineered strains (*Ec-ΔY*-PPH, *Ec-ΔP*-PPH and *Ec-ΔY-ΔP*-PPH). Indeed, *Ec-ΔY*-PPH and *Ec-ΔP*-PPH strains produced 15.04 and 17.76 mM of 3-HP, respectively, whereas deletion of both *ygfH* and *prpC* genes in the *Ec-ΔY-ΔP*-PPH strain increased the 3-HP production from 9.23 to 24.14 mM, i.e. 2.6-fold. This underscores the importance of redirecting the metabolic fluxes through the designed pathway by disrupting processes that compete for propionyl-CoA.

## Discussion

In this work, recombinant *E*. *coli* strains were engineered to express the key enzymes (PACD, PCT and HPCD) of the propionyl-CoA pathway for 3-HP production. Recombinant *E*. *coli* could produce 3-HP when cultured in a propionate-containing medium. Considering that propionyl-CoA is the key intermediate in this pathway and that propionate could be produced by some types of microorganisms [47], including the popular engineering host *E*. *coli* [16], to produce 3-HP, the propionyl-CoA pathway is a promising alternative route to the commonly reported pathways that use glucose or glycerol as the carbon source [6, 12].

If no propionate was fed in the medium, 3-HP could not be detected in the broth of the recombinant strains during the whole period of cultivation (0–25 h). This result suggests that the propionyl-CoA pool of the host strain was not sufficient to produce a detectable amount of 3-HP. In contrast to glucose or glycerol, propionate is not a usual and economical carbon source for bio-based product synthesis. As propionate itself is a biological product and its biosynthesis pathways in *Propionibacterium* [47] and *E*. *coli* [16] have been well established, further efforts are required to improve the intracellular propionyl-CoA pool by introducing a propionate/propionyl-CoA synthesis pathway into the recombinant strains mentioned above.

The intracellular propionyl-CoA pool might also be enhanced by inhibiting the endogenous propionyl-CoA catabolism, so we disrupted the metabolic pathways diverted to the MC cycle and/or the TCA cycle (Fig 1). Based on previous reports that the conversion of propionyl-CoA to target products could be improved by the deletion of the *ygfH* gene encoding propionyl-CoA:succinate CoA-transferase [26, 48] or of the *prpC* gene encoding 2-methylcitrate synthase [29, 48], the effect of deleting these genes on the 3-HP production was examined (Fig 5).

The results indicate that the effect of *ygfH* gene deletion on the 3-HP titer was similar to that of *prpC* gene deletion, and the combination of the *ygfH* and *prpC* gene deletions increased the 3-HP titer, exhibiting an efficiency higher than that of either single gene deletion. However, in a previously reported work on the accumulation of a propionyl-CoA derived product, poly(3-hydroxybutyrate-co-3-hydroxyvalerate), in *E*. *coli* [48], deletion of the *prpC* gene did not significantly increase the 3-HV (3-hydroxyvalerate) fraction, while deletion of the *ygfH* gene significantly increased the 3-HV fraction in the copolymer. By comparing our work to that of Chen et al [48], there were differences in the effects of *ygfH* and *prpC* gene deletion on the product accumulations, suggesting that the metabolic flux from propionyl-CoA to the MC or TCA cycle in *E*. *coli* was product-dependent.

By culturing these recombinant strains in propionate-containing medium, the conversion yields of propionate to 3-HP were typically in the range of 3% to 15%, implying that the native metabolism of propionate detracts propionyl-CoA from the desired 3-HP biosynthesis. By disrupting the genes for key enzymes of the competing metabolic pathways, *ygfH* and *prpC*, the 3-HP yield on propionate for *Ec*-ΔY-ΔP-PPH increased to 35.4%, not a very high value yet, suggesting that there are other unidentified propionyl-CoA metabolic pathway(s) in the *E*. *coli* host. Propionyl-CoA carboxylase (PCC), an enzyme that can effectively convert propionyl-CoA to (2*S*)-methylmalonyl-CoA, has been reported in many species [43, 49], although not in *E*. *coli*. Considering that the carboxylations of acetyl-CoA and propionyl-CoA are often shown to be catalyzed by a single ATP-dependent enzyme [43, 50], the function of propionyl-CoA carboxylation in *E*. *coli* might be applied by acetyl-CoA carboxylase (ACC). However, ACC is essential for the central metabolism of the host cell and can not be deleted. In addition, the acetyl-CoA:short-chain fatty acid CoA-transferase in *E*. *coli* might act as the isoenzyme of YgfH because of the broad substrate specificity of bacterial CoA transferases for short-chain acyl-CoA thioesters [18, 38], and the intracellular propionyl-CoA concentration would be reduced by the reaction of CoA transferase even in the ΔygfH strain. Thus, further down-regulating the propionyl-CoA catabolic flux and increasing the intracellular propionyl-CoA pool by, e.g., introducing genes for the synthesis of propionyl-CoA in the host might contribute to the future improvement of the 3-HP yield.

In conclusion, a PACD gene from *Candida rugosa* was heterologously expressed in *E*. *coli* and its effect on 3-HP production was demonstrated. By overexpressing PCT and HPCD along with PACD, 3-HP production improved six-fold due to the strengthened metabolic flux to 3-HP. The presence of glucose in the medium and the use of an appropriate temperature ensured that there was a sufficient energy source and optimal enzyme thermostability, which contributed to higher 3-HP titers. Furthermore, disruption of the competing propionyl-CoA metabolic pathways by deleting the *ygfH* and *prpC* genes resulted in a 2.6-fold improvement in the 3-HP titer. It is expected that the 3-HP yield of the recombinant strains could be increased by further minimizing the carbon flux to the other metabolites and balancing the activities among the targeted enzymes. To the best of our knowledge, this is the first report on the biosynthesis of 3-HP in a recombinant strain via the propionyl-CoA pathway.

## Supporting Information

S1 Table Primers used in the study. The primer sequences and the PCR conditions are shown. The restriction enzyme sites in the primer for subsequent cloning are underlined.
(DOCX)
