# Supplementary material for: Production of 3-Hydroxypropionic Acid via the Propionyl-CoA Pathway Using Recombinant Escherichia coli Strains
Source: PLoS One. 2016 May 26;11(5):e0156286. doi: 10.1371/journal.pone.0156286 (PMC4882031; doi:10.1371/journal.pone.0156286)
Supplement: S1 Table — The primer sequences and the PCR conditions are shown. The restriction enzyme sites in the primer for subsequent cloning are underlined. (DOCX) [file pone.0156286.s001.docx]

**S1 Table**

**Primers used in the study.** The primer sequences and the PCR conditions are shown. The restriction enzyme site in primers for subsequent cloning was underlined.

| Primer name | Sequence (5’→3’) | Base pair (bp) | PCR conditions |
| --- | --- | --- | --- |
| *pacd-Nde*Ι-FP | GGTACTCCATATGTCGATTAAGGACG | 26 | 94°C 5 min;  94°C 40 s, 50°C 40s, 72°C 2 min 30 s, 28 cycles |
| *pacd-Xho*Ι-RP | CCGCTCGAGTTACAACTTGGCCAAC | 25 |  |
| *pct- Nde*Ι-FP | CCAATCTCCATATGAGAAAAGTAGAAATCATTA | 33 | 94°C 5 min;  94°C 40 s, 50°C 40s, 72°C 2 min 30 s, 30 cycles |
| *pct-BamH*Ι-RP | TATGGATCCTTATTTTTTCAGTCCCATGGGA | 31 |  |
| *hpcd*- *Nde*Ι-FP | CCAATCTCCATATGAGTGAAGAGTCTCTG | 29 | 94°C 5 min;  94°C 40 s, 55°C 40s, 72°C 2 min 30 s, 30 cycles |
| *hpcd-BamH*Ι-RP | TATGGATCCAGATCGCAATCGCTCGTG | 27 |  |
| YgfH-FP | GGAGTAAAAATGGTTGGTCATTAATCCCTGCGAACGAAGAAACTCAGTGGGTGTAGGCTGGAGCTGCTTC | 70 | 94°C 5 min;  94°C 40 s, 54°C 40s, 72°C 2 min 20 s, 30 cycles |
| YgfH-RP | ATTACGGTGTAAGTCGAAGACGTGGCTAAGATCGTGGTGAATATGTCCGCATTCCGGGGATCCGTCGACC | 70 |  |
| PrpC-FP | ATCTCGACCCTACAAATGATAACAATGACGAGGACAACATGAGCGACACAGCTGGAGCTGCTTCGAAGTTC | 71 | 94°C 5 min;  94°C 40 s, 54°C 40s, 72°C 2 min 20 s, 30 cycles |
| PrpC-RP | TTTGCTCTCCCACATCACCGTTTCCAGGCGATCGGCGATGTTGTACATCTATTCCGGGGATCCGTCGACC | 70 |  |
